# Foodomic-Based Approach for the Control and Quality Improvement of Dairy Products

**DOI:** 10.3390/metabo11120818

**Published:** 2021-11-29

**Authors:** Rubén Agregán, Noemí Echegaray, Asad Nawaz, Christophe Hano, Gholamreza Gohari, Mirian Pateiro, José M. Lorenzo

**Affiliations:** 1 Centro Tecnológico de la Carne de Galicia, Adva. Galicia n° 4, Parque Tecnológico de Galicia, San Cibrao das Viñas, 32900 Ourense, Spain; rubenagregan@ceteca.net (R.A.); noemiechegaray@ceteca.net (N.E.); mirianpateiro@ceteca.net (M.P.); 2 Jiangsu Key Laboratory of Crop Genetics and Physiology, College of Agriculture, Yangzhou University, Yangzhou 225009, China; 007298@yzu.edu.cn; 3 Co-Innovation Center for Modern Production Technology of Grain Crops, Yangzhou University, Yangzhou 225009, China; 4 Laboratoire de Biologie des Ligneux et des Grandes Cultures, INRA USC1328, Orleans University, CEDEX 2, 45067 Orléans, France; hano@univ-orleans.fr; 5 Department of Horticulture, Faculty of Agriculture, University of Maragheh, Maragheh 83111-55181, Iran; gohari.gh@maragheh.ac.ir; 6 Área de Tecnología de los Alimentos, Facultad de Ciencias de Ourense, Universidad de Vigo, 32004 Ourense, Spain

**Keywords:** transcriptomics, proteomics, metabolomics, food safety, food fraud, transformation processes

## Abstract

The food quality assurance before selling is a needed requirement intended for protecting consumer interests. In the same way, it is also indispensable to promote continuous improvement of sensory and nutritional properties. In this regard, food research has recently contributed with studies focused on the use of ‘foodomics’. This review focuses on the use of this technology, represented by transcriptomics, proteomics, and metabolomics, for the control and quality improvement of dairy products. The complex matrix of these foods requires sophisticated technology able to extract large amounts of information with which to influence their aptitude for consumption. Thus, throughout the article, different applications of the aforementioned technologies are described and discussed in essential matters related to food quality, such as the detection of fraud and/or adulterations, microbiological safety, and the assessment and improvement of transformation industrial processes (e.g., fermentation and ripening). The magnitude of the reported results may open the door to an in-depth transformation of the most conventional analytical processes, with the introduction of new techniques that allow a greater understanding of the biochemical phenomena occurred in this type of food.

## 1. Introduction

The current methodology available for the control of industrial processes in feeding or the identification of biological contaminants in food has limitations [1]. For this reason, analytical approaches are gradually moving towards more innovative and interdisciplinary methodologies, leaving behind those considered conventional. The use of ‘omic’ technologies for the analysis of food provides results of a higher analytical quality and can be used according to the approach that the researcher wants to give to the study. Thus, we can find genomic (study of the genome), transcriptomic (study of the transcriptome), proteomic (study of the proteome), and metabolomic (study of the metabolome) technologies, capable of analyzing and describing food products, generating data of the differential expression of genes, transcripts, proteins, and metabolites, respectively [2]. On the other hand, for a better and more exhaustive characterization of the complexity of the biological system of a food, omic technologies can be used at a multilevel mode, which is known as the multi-omic approach, perhaps providing a clearer representation of what occurs to matrix level [3].

The application of these technologies in food science and technology is called ‘foodomics’ and is addressing problems related to food safety and quality control, gradually replacing, if not complementing, already established methodologies [4]. The first time the term foodomics was proposed was in 2009 by Alejandro Cifuentes [5]. Since that time, omic-based techniques have contributed to providing solutions, helping to understand the impact that food and its ingredients have on our body at the molecular level.

Different research studies have recently addressed the use of foodmics for the analysis of food components in search of continuous improvement of the food presented to the consumer at the point of sale. This article presents an updated review of the ability of transcriptomic, proteomic, and metabolomic technologies for food quality control in dairy products. These omic technologies are revolutionizing the understanding of the biological structure of these foods, contributing to the detection of fraud and food safety. In addition, in the present review, it is emphasized how these technologies can help improve the quality of dairy products through the study of the changes produced in their matrix due to the different stages of transformation in the industry, generating the necessary knowledge to influence in the final characteristics of the product.

## 2. Description and Application of Transcriptomics, Proteomics and Metabolomics in Food Analysis

### 2.1. Transcriptomics

The transcriptome can be defined as the complete set of RNA transcripts produced by the genome at a given time, connecting the phenotype with the information encoded in the DNA. The study of this transcriptome is known as transcriptomics and is based on the differential identification of genes expressed under different conditions [6], helping to understand the functional elements of the genome and showing the molecular constituents of cells [7]. Nucleic acid analysis according to transcriptomic technology is based on exploiting the substrate specificity of DNA-metabolizing enzymes, such as restriction endonucleases, DNA polymerases, reverse transcriptases, or DNA ligases, and using the base-pairing properties that promote spontaneous hybridization of complementary polymeric products [8].

Transcriptome can be addressed through three techniques basically: real-time reverse transcription (RT)-PCR, microarray, and next-generation RNA sequencing (RNA-seq) [9]. When the aim of the study is focused on certain specific genes, real-time RT-PCR can be a suitable technique by allowing the detection of small differences in gene expression. In addition, it highlights for its wide dynamic range, homogeneity, and a scarcely existing variation between trials [10]. Despite these positive aspects, its application in biological samples is not exempt of problems. Thus, for example, there is an inherent variability of ARN, another related to extraction protocols, and different reverse transcription and PCR efficiencies. For these reasons, a precise normalization method is necessary to address these vulnerabilities. However, this normalization brings a headache for analysts [11]. All these drawbacks led to the implantation of other techniques, such as microarrays and RNA-sequencing (RNA-seq), which currently dominate the study of the transcriptome. The first is based on the quantification of a set of predetermined sequences and the second on the capture of all sequences by means of a high-throughput sequencing [9]. The microarrays technique is a very interesting option for the evaluation of most of the transcriptome of a sample. There are already predesigned microarrays for microbiological analysis able to assess the transcriptome of foodborne pathogens such as *Escherichia coli* [12]. On the other hand, the RNA-seq has become a reference technique for transcriptomic studies over the years, providing more complete information regarding real time RT-PCR and microarrays techniques. This technique can be used for the exploration of new classes of RNA. Furthermore, it is possible to detect point mutations in the sequence of certain transcripts, together with unknown splice variants [6].

**Figure 1:**
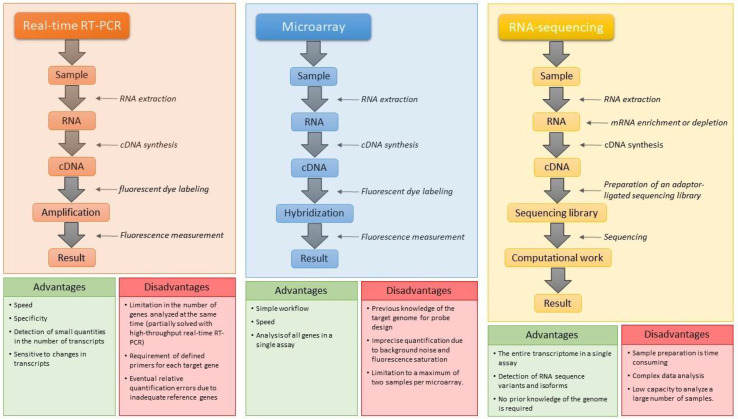
Brief flow diagram of the technologies used in transcriptomics with their advantages and disadvantages. cDNA: complementary DNA; mRNA: messenger RNA. Data from Hugget et al. [11], Kaliyappan et al. [13], Lamas et al. [6], and Stark et al. [14].

The pros and cons of these technologies and their workflow are depicted in Figure 1.

### 2.2. Proteomics

Proteomics is another technique that belongs to the group of so-called omic techniques that deals with the large-scale of proteins in biological systems at a given moment in time. This temporal specificity is due to the dynamicity of the proteome in response to different stimuli from its environment [15]. On the other hand, the proteome is nothing more than the set of proteins encoded by the genome, including all isoforms and modifications, as well as the interactions between them, their structures, and higher-order complexes [16]. The term proteomics (PROTEins expressed by the genOME) was first coined in the early 1990s by Marc Wilkins and coworkers and was presented during the first proteomic conference in the Italian city of Siena in 1993, although the discipline as such was borne some time earlier, in the late 1970s, when Norman G. Ander and N. Leigh attempted to unlock the human genome by identifying its respective proteins.

The techniques used in protein analysis are extensive and have evolved over time. Those that are now considered conventional, such as the enzyme-linked immunosorbent assay (ELISA) and western blotting, have given way to other more modern and advanced techniques. In the 1970s, sodium dodecyl sulphate (SDS) electrophoresis was introduced as a technique for the separation and characterization of proteins, to be later complemented with the isoelectric focusing (IEF) technique in search of an improved resolution [17], known as two-dimensional gel electrophoresis (2-DGE). Variations of this technique were emerging, such as the use of immobilized pH gradients (IPGs) and two-dimensional difference gel electrophoresis (2D-DIGE), which uses differential labeling of protein samples with fluorescent tags to enhance sensitivity and reproducibility [18]. On the other hand, the appearance of mass spectrometry (MS) technique was a real revolution in proteomic analysis by allowing a more exhaustive and rapid characterization of proteomes. This technique uses a wide range of instruments that cover many possible applications, allowing the analysis of complex protein samples and sequences, the study of protein-protein interactions, and the identification of post-translational modifications [19].

Today, gel-based approaches are every time less used, being gradually replaced by other approaches that provide quantitative data on the variations occurred in the sample proteome. This is the case of the so-called shotgun approach, based on the enzymatic digestion of intact protein and its subsequent fractionation and characterization using the liquid chromatography coupled with tandem mass spectrometry (LC-MS/MS) technique [20]. In this way, hundreds of thousands of peptides species can be resolved in a space of time of just few hours [21]. This approach is an excellent method of comparative analysis between samples that also allows their previous labelling with isotopes, which gives rise to different analysis methods, such as stable isotope labeling with amino acids in cell culture (SILAC), stable isotope labeling in mammals (SILAM), isotope affinity tagging (ICAT), isobaric tags for relative absolute quantification (iTRAQ), tandem mass tag (TMT), and dimethyl labeling [20]. The shotgun approach enables the massive quantification of thousands of unknown proteins. However, a targeted analysis towards a section of the proteome can be very interesting when it is previously known, avoiding the exhausted work of tracking huge amounts of proteins. Targeted proteomics, which has been growing so widely in the last decade, allows to work with a limited fraction of the proteome and check possible changes in its expression, measuring a large set of samples with high precision and reproducibility [22].

### 2.3. Metabolomics

Metabolomics is a fundamental piece within the set of omic techniques that comprise the foodomic approach and that, as in the case of transcriptomics and proteomics, can be applied in the study of aspects related with the areas of food and nutrition. Metabolomics can be defined as the systematic identification and quantification of all metabolites in an organism or specific biological sample [23]. As a result, a profile of small molecules from cellular metabolism is generated and that can directly reflect the result of the network of biochemical reactions that occur in a biological system [24], providing essential information on the state of such system. However, the variability of compounds present below a size of 1500 Da can be enormous, making metabolomic analysis difficult. Food matrices are a clear example of this, including carbohydrates, lipids, proteins, amino acids, amines, steroids, phenolic compounds, carotenoids, alkaloids, or volatile compounds, among others. Therefore, for a comprehensive study of the metabolome of complex biological systems such as dairy products, metabolomics needs to be complemented with other analytical approaches [25].

Nowadays, metabolomic analysis is performed primarily through two techniques, nuclear magnetic resonance (NMR) spectroscopy and MS. The first is a technique specially focused on high-performance analytical studies by being a more cost-effective and less time-consuming technology than MS. In addition, it offers high reproducibility. On the other hand, NMR is to date the unique suitable technique for the quantification or structural identification of unknown compounds [26]. In contrast, available databases for analysis of NMR spectra cover only a fraction of the relevant compounds. In addition, the low sensitivity of the technique can make it difficult to identify compounds. Nevertheless, technology capable of partially solving this deficiency is being implemented, such as NMR spectrometers with ultra-high-field magnets operating at H resonance frequencies of 1.2 GHz or higher and the use of hyperpolarization, which has been reported to enhance sensitivity with even higher potential [27]. Conversely, MS-based metabolomic analysis obtains a better rating for this parameter, also highlighting for its speed and wide dynamic range. However, as any technique, it is not exempt of problems that can limit its application, such as the difficulty in detecting metabolites at trace levels. On the other hand, the constant improvement of sample processing methods, the coupling of chromatographic technology (e.g., liquid and gas chromatographies), and the already mentioned high sensitivity favor the obtaining of massive amounts of information, which hinder their subsequent processing. Later, the identification of the metabolites is another challenge again due to problems, such as the large amount of data obtained after peak alignment and the poor databases available. Although an improvement in the technology used is expected to gradually remedy these deficiencies [28]. Despite these disadvantages, MS is considered an ideal technique, with promising potential in metabolomic research.

## 3. Application of Foodomics for Quality Control in Dairy Products

### 3.1. Microbiological Contaminants. Detection of Pathogens and Their Toxins

The especially nutrient-rich matrix of dairy products is an excellent culture medium for the growth of hazardous microorganisms, such as *Brucella abortus*, *Brucella melitensis*, *Campylobacter jejuni*, *Escherichia coli*, *Listeria monocytogenes*, *Mycobacterium bovis*, *Mycobacterium tuberculosis*, *Salmonella*, *Staphyloccocus aureus*, and *Yersinia enterocolitica*, which can seriously deteriorate the health of certain groups, such as children, pregnant women, and elderly [29]. Transcriptomics could be very useful in predicting the activity of these pathogenic microorganisms in dairy products. As previously mentioned in this review, it is possible to understand microbial behavior in different exogenous conditions through the analysis of RNA molecules, since the food matrix plays an important role in the expression of genes involved in activities, such as growth, survival, or level of virulence towards the host [30]. Cretenet et al. [31] used the transcriptomic technology to assess the extent to which a cheese matrix could modulate the expression of virulence genes in *Staphylococcus aureus*. After studying the behavior of the bacterium without and in the company of the endogenous bacterium *Lactococcus lactis*, a dominant species in the cheese ecosystem, the researchers observed that, depending on the presence or absence of the latter bacterium, there is an upward or downward production of certain enterotoxins. Thus, for example, the expression of the sea toxin was slightly favored by the presence of *Lactococcus lactis*, on the contrary that the *sec4* toxin, which was down-regulated. On the other hand, the low pH caused by the presence of the endogenous bacterium was one of the factors behind the down regulation of the accessory gene regulator (*agr*) system, a key regulator of bacterial virulence. Therefore, in view of these results, transcriptomic studies might open new ways for the development of novel prevention strategies against the main foodborne pathogens.

Instead of trying to predict the possible virulence of certain species in food, its early detection may be more interesting to prevent any possible episode or event of an infectious nature. The study of the proteome of microorganisms can be an efficient strategy for the rapid detection of pathogens in food samples, as recently published studies show. The technique matrix-assisted laser desorption/ionization and subsequent analysis of the ions produced by time-of-flight (MALDI-TOF) seems to have fulfilled the expectations of the researches, providing satisfactory results. The target of applying this technique is the obtaining of a characteristic protein fingerprint of each bacterium at a given time and determined physiological condition. This specific protein profile might be used for the unequivocal detection of pathogens in food matrices [32]. Karasu-Yalcin et al. [33] used this technique to find the presence of the microorganism *Listeria monocytogenes*, a particularly dangerous bacterium, in liquid culture media. Incubation for only 18 h of an initial inoculum of 3 × 101 cfu/mL in sterile BHI broth was enough for its detection. However, when tested on a real product such white cheese, the method did not provide reliable identification. The same result was reported in another recent study for the same bacterium. Pyz-Łukasik et al. [34] were also not able to achieve a satisfactory identification by means of an analysis with MALDI-TOF MS in artisan cheeses. Jadhav et al. [35] reached the identification of the genus *Listeria* in isolates of the bacterium from dairy sources, but not the species, identification of which was found to be influenced by the culture conditions, suggesting the need to standardize this step for protocols with MALDI-TOF MS. On the contrary, this technique was effective for rapid identification of pathogens of the *Enterobacteriaceae* family such as *Escherichia coli*, isolated from both milk and dairy products (cottage cheese and butter) [36]. These disparate results for the recognition of microorganisms suggest the need to expand the mass spectral databases, especially for *Listeria monocytogenes* strains present in dairy products such as cheese. Analysis of the metabolite profile generated by microorganisms can be another effective way to confirm their presence in food. Specifically, it is possible to identify pathogen-specific biomarkers by untargeted metabolomics as showed by Jadhav et al. [37] after using gas chromatography coupled with mass spectrometry (GC-MS) for the characterization of *Escherichia coli*, *Listeria monocytogenes*, and *Salmonella enterica* bacteria. The application of the technique in a nutritious liquid medium containing spiked meat samples resulted in the finding of putative biomarkers related to these bacteria, highlighting the identification of sugars, fatty acids, amino acids, nucleosides, and organic acids. Additionally, the study included a proteomic analysis using MALDI-TOF MS as a complement to the metabolomic analysis to contrast and corroborate the data obtained, demonstrating the complementarity of both omic technologies for the detection of pathogens in food samples. These new analytical approaches have the ability to significantly reduce the time of analysis and produce results in a short space of time compared to conventional microbiological methods that can take up to a week to confirm the presence of pathogens. This is especially relevant in dairy industries with large processing capacity that output a significant number of products.

The foodomic approach can help in the detection of mycotoxins in food. These toxins of fungal origin are secondary metabolites that may cause serious health problems once ingested, since they have been reported to possess carcinogenic, immunosuppressive, hepatotoxic, nephrotoxic, and neurotoxic effects [38]. Dairy products figure as a major source of distribution for some of these toxins, especially aflatoxins, but also fumonisin, ochratoxin A, trichothecenes, zearalenone, T-2 toxin, and deoxynivalenol [39]. Specifically, an upward risk of finding these compounds in cheese products has been reported as a consequence of the metabolism of producing fungal species [40,41]. Hence, it is important to find new tools that enhance their detention at any point in the food chain. The omic-based approach has shown to be useful in the mycotoxic analysis of milk from farm animals [42,43]. In the same way, it might also be applicable to already processed products such as cheeses.

### 3.2. Authenticity Assurance. Struggle against Food Fraud and the Presence of Undeclared Allergens

The lack of equivalence between the real product and the one specified on the label is considered fraud towards the consumer [44] and may even constitute a crime. Therefore, the food industry must always be vigilant to protect the integrity of food and consumer rights. To date, a number of techniques including chromatography, CE, and spectroscopy have been used successfully for the detection of food fraud. However, the appearance of recent omics technologies together with their techniques are prevailing over other more conventional methodologies due to their better sensitivity, precision, multiplexing and quantitative accuracy [45].

One of the most common fraudulent practices faced by the dairy industry is the substitution of a declared type of milk for another that is economically more profitable [46]. This adulteration of milk also affects the products made from it. In particular, this fraud is especially concerning in the cheese production, since many owe their quality distinction in a great extent to the exclusive origin of the milk used. Both the proteome and metabolome of the cells of the dairy animal have been studied to detect contaminations. These studies are based on finding an unequivocal and reliable fingerprint with to correctly identify the product.

The addition of cow’s milk to others of different species is one of the most common adulterations due to its low price and high availability throughout the year. These fraudulent procedures also modify the nutritional profile, and what is worse, they can expose a part of the population sensitive to cow’s milk proteins to a health problem. Therefore, the application of precise detection methods is required. The standard procedure for the identification of cow’s milk in goat’s or ewe’s milk is based on the presence of γ2 and γ3 casein bands in an IEF gel, but certain limitations, such as the impossibility of detecting adulterations below 5% using common staining procedures or the presence of interfering bands that can give false positives, have led to the search for alternative methods based on spectrophotometry [47].

**Figure 2:**
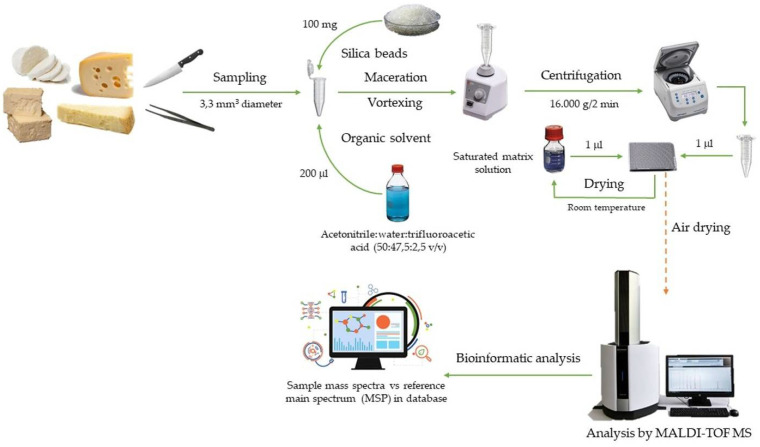
Analytical workflow using MALDI-TOF MS for detection of possible cheese fraud.

As already discussed in the previous section, the MALDI-TOF MS technique can provide a comprehensive knowledge of the cellular proteome, and just as it can help to identify bacteria, it can also do so with the species of the milk analyzed by finding characteristic protein markers of each one. Through these specific markers, many other differentiations can also be established that help identify fraud in dairy products. Some of the most relevant applications of this proteomic technique in assuring the authenticity of cheese products are reflected in Table 1. The low amount of sample required and the possibility of analysis of heterogeneous samples (Figure 2), as well as the speed showed, make the MALDI-TOF MS technique as one of the most promising in routine analysis for the detection of fraud in the dairy industry [48].

Contamination of dairy products with cow’s milk (e.g., yogurts or cheeses) made with milk from different species could lead to problems of hypersensitivity to certain proteins, such as caseins, α-lactalbumin, and β-lactoglobulin, proteins that frequently trigger an immune response to those individuals allergic to cow’s milk, although actually all proteins can be potential allergens, regardless of whether they are at trace levels [55]. These contaminations can be accidental within the same industry, but whatever their origin it is essential to detect them. As discussed above, one possible way to detect these proteins is by identifying the animal origin of the raw material. However, these proteins can also be individually identified. Ji et al. [56] used a targeted proteomic-based on multiple reactions-monitoring (MRM) to confirm the presence and quantifying the α_s1_-casein, α-lactalbumin, and β-lactoglobulin proteins in different food products. Five and 3 peptides from these proteins were selected using MALDI tandem time-of-flight (TOF/TOF) MS technology to serve as a fingerprint in their detection and subsequent quantification, respectively. The results obtained were satisfactory enough as to validate the method, noting a potential application as a routine analysis in the detection of allergens in food. Similarly, Qi et al. [57] found a fingerprint for the detection of α_s1_-casein, β-casein, and κ-casein in baked goods, composed of 2 or 3 peptides for each protein. Subsequently, the application of targeted proteomics using a MRM approach allowed to quantify these proteins in the corresponding food matrices.

Metabolomics also provides distinctive markers for each product with which to corroborate its identity. A factor such the breed will produce a characteristic metabolomic profile in the milk, extendable to products made with it. Similarly, extrinsic factors, such as diet, geographical location, processing, or storage will have the same effect [58]. Correctly selected metabolites as biomarkers may be an excellent platform for the determination of adulterations in milk and dairy products. Rocchetti et al. [59] studied to stablish a chemical fingerprint to discriminate between Grana Padano Cheeses with a protected designation of origin (PDO) and without it. An untargeted metabolomic approach using ultra-high pressure liquid chromatography coupled with quadrupole time-of-flight mass spectrometry (UHPLC-QTOF MS) allowed select the markers that best characterized the cheeses, both with and without quality seal. Any variation from the standard profile found could means fraud. Similarly, Salzano et al. [60] used GC-MS to create a fingerprint to aid in the authentication of mozzarella cheeses with PDO. In this way, 185 metabolites were consistently detected in buffalo milk and cheese, creating a library of compounds that was able to discriminate among products with and without PDO. These studies make it clear that MS-based approaches are a powerful tool for creating a protection mark to help detect fraud in dairy products. NMR technique can also be useful to draw lines of differentiation between dairy products as it has been shown to be effective in discriminating milk samples affected by environmental factors [61]. However, its use seems more aimed at the analysis of homogeneous liquid foods due to the problems that heterogeneous foods such as milk present. Complex matrices with an emulsified colloid consisting of small globules of fat suspended in water make it difficult to obtain a well-resolved proton nuclear magnetic resonance (H-NMR) spectrum [62].

Information related to the origin of the product contributes to improve food traceability, thus increasing overall quality. Consumers are increasingly demanding more information about each stage of the food chain and, in this sense, both metabolomic and proteomic-based approaches are able to provide an appropriate level of detail. In the near future, omic techniques might offer a competitive advantage to address food safety concerns and nutritional problems that affect the consumer and give the product maximum added value. On the other hand, traceability can provide information not only on the origin but on other factors that also affect the final quality, such as the transformation processes, altering nutritional and organoleptic properties of foods [63]. This is especially relevant in dairy products, such as yogurt, kefir, or cheese, with market value depending on certain extrinsic factors, among which fermentation and/or maturation should be highlighted.

### 3.3. Evaluation of Industrial Processes for the Improvement of Dairy Products

**Figure 3:**
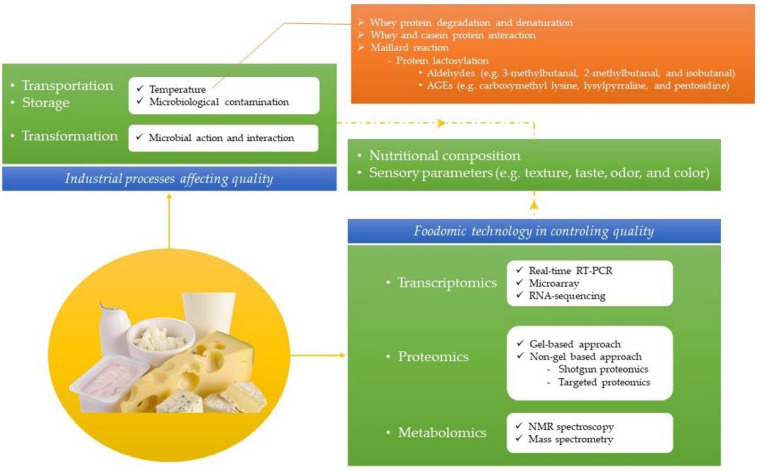
Foodomics for quality control in the dairy industry.

The industrial processes or operations of dairy products can have a negative effect on their quality, acting as true external agents. One of the basic operations that may cause the most damage to the product is heat treatment, damaging its aptitude for consumption. Different chemical processes derived from the heating of milk have been reported to have harmful consequences in its matrix, affecting nutritional and sensory properties (Figure 3).

Proteins are especially vulnerable to heat and are involved in the main reactions caused by thermal treatments of milk, including protein denaturation and aggregation and Maillard reactions (non-enzymatic browning) [64]. Proteomics has recently been used for the in-depth study of these changes, enhancing the understanding of the reactions occurred in the matrix of milk and dairy products with the aim of minimizing the derived effects on quality. In a study on the influence of heat treatment intensity on goat’s milk proteins, a proteome modification of almost 20% was observed. Chen et al. [65] used label-free quantification to find a differential expression of up to 29 proteins due to the thermal treatments applied to milk. The results showed that long treatment times were more damaging with milk proteins than short treatments. A treatment with low temperature for a long period of time (65 °C, 30 min) produced the greatest changes in the proteome, revealing modifications in the nutritional profile. Temperatures above 85 °C would cause a cleavage of the disulfide bonds of β-lactoglobulin to generate free sulfhydryl groups and sulfides, affecting flavor. A progressive increase in temperature above this value would lead to the degradation of this protein and to a decrease in the content in milk and therefore in the dairy product made with it. In general, there would be a denaturation of the serum fraction, as well as a covalent interaction between whey and casein micelles. Although in certain cases, this is an advantage such as in the production of yogurt, in others, it could certainly be a disadvantage. Hence, it is important to control the degree of denaturation of the protein β-lactoglobulin, since it might affect the functionality of the food [66]. Heat damage to β-lactoglobulin may to cause changes in its structure. Specifically, a proteomic-based approach revealed that reactions of lactosylation, carboxymethylation, formylation of lysine and the N-terminus, glycation of arginine, oxidation of methionine, tryptophan, and cysteine, oxidative deamination of the N-terminus, and the deamidation of asparagine and glutamine are found common during the thermal treatment of milk [67].

The Maillard reaction in milk and dairy products produces a loss of both organoleptic and nutritional quality, which will be acuter the more intense the heating. The initial reaction of lactose with milk proteins (mostly lysine), known as protein lactosylation and induced by a high temperature, originates a cascade of reactions that produce compounds of a wide variety, most of them responsible for abnormal tastes and odors, such as 3-methylbutanal, 2-methylbutanal, and isobutanal [68]. In addition, other compounds such as advanced glycation end products (AGEs), including carboxymethyl lysine, lysylpyrraline, and pentosidine, which have been linked to health problems [69]. Milkovska-Stamenova and Hoffmann [70] studied the presence of AGEs in cow’s milk with nano ultra-high performance liquid chromatography couple with electrospray ionization tandem mass spectrometry (nUPLC-ESI-MS/MS). Up to 14 different types of these protein-derived compounds present in the raw product or during its processing/transformation were identified. In total, the modification of 132 peptides by at least one AGE studied was reported, suggesting the power of the proteomic approach for the study of these or related changes in the milk, before, during, and after its processing and/or transformation. In this line, Meltretter et al. [71] found in the MALDI-TOF MS technique a fast and reliable way to measure the damage in milk and dairy products by thermal processes. The increasing intensity of the treatment in the milk produced additional signals with a mass shift that was associated with the participation of 2 lactose molecules in the reaction. The increase in the participation of lactose molecules was even greater in the treatment of infant formulas, involving 3 or 4 molecules, which reflects the high glycation degree when the heating is excessive in this type of products. MS-based research has linked the intensity of heat applied in dairy products to the accumulation of lactose and hexose adducts. Hence, the degree of lactosylation and hexosylation might be used as a marker of the strength of the thermal treatment in this type of products [72]. Chandra Roy et al. [73] found a similar correlation for goat’s milk when subjected to increasing treatment intensities. The number of proteins initially identified in the whey fraction by sodium dodecyl sulphate polyacrilamide gel electrophoresis (SDS-PAGE) and LC-MS/MS decreased as the intensity of heat treatment increased. Of the 202 initial proteins, 194, 186, and 178 remained after the pasteurization, ultra-high temperature (UHT), and spray-drying treatments, respectively. Similarly, lactosylation sites increased from 16 in raw untreated milk to 20 in pasteurized milk, 25 in UHT milk, and 27 in spray-dried milk. Precisely, in this last type of product, the current treatment process considerably affects serum proteins. Liu et al. [74] reported a retention of 70–85% of lactoferrin and immunoglobulins when the treatment intensity is reduced during the production process of milk powder.

Storage is another critical point in the manufacture of some dairy products that can remarkably affect the quality of the product. Fluctuations in the temperature during the preservation of milk powders, including infant formulas, can lead to the appearance of non-enzymatic browning phenomena. Glycolytic activity has been reported in these products even at refrigerated temperatures. Milkovska-Stamenova et al. [72] found an increase in the level of lactosylated molecules in an infant formula after 1 year of storage at refrigerated temperature. The characterization of the sample proteome using a gel-based approach, together with LC-MS/MS, showed a significant increase in the degree of lactosylation (2 to 4 times higher than that found for UHT milk in the same study) for α-lactalbumin, glycosylation-dependent cell adhesion molecule 1, bovine serum albumin, xanthine dehydrogenase, platelet glycoprotein 4, κ-casein, β-casein, lactotransferrin, and polymeric immunoglobulin receptor. The application of proteomics in the study of the evolution of these reactions displayed that even at low amounts, the mere presence of lactose is enough to trigger a non-enzymatic browning in this type of products during storage, as well as to promote the generation of lactosylation residues, favoring changes in color and pH [75].

Metabolomics can also contribute with solutions in the loss of quality during the processing of milk and dairy products. The study of the modification of the vitamin, organic acid or fatty acid profile may help to modulate the treatment and optimize the choice of the most suitable process for maintaining food safety with the minimum loss of properties. The heat applied during the thermal treatment of milk is once again the protagonist, affecting other compounds besides proteins. Caboni et al. [76] observed differences in the fatty acid profile and other compounds due to the heat treatment of a raw ewe’s milk for the elaboration of a PDO Fiore Sardo cheese, although in general terms the changes experienced were minimal compared to those reported as a result of the ripening process or the parameter of seasonality. Another example where heat can play a determining role in the differential formation of compounds is during the fermentation stage. This was confirmed by Yang et al. [77] during the elaboration of yogurts made with different fermentation temperatures. An untargeted approach showed significant changes in amino acid and fatty acid profiles, in addition to other quality parameters, such as the water holding capacity, texture, and flavor. Other compounds, such as lipid-like molecules, organic acids and their derivatives, and organoheterocyclic compounds, were also found to be notably affected. Trimigno et al. [78] also noted that the previous heat treatment of milk can affect the activity of starter cultures in the yogurt and influence the production of metabolites. Monitoring the fermentation stage by H-NMR metabolomics provided a comprehensive and quantitative chemical description of what happened during this time in the milky matrix. It was observed that the differential thermal treatment affected the protocooperation of *Lactobacillus delbrueckii* subsp. *bulgaricus* and *Streptococcus thermophilus* (bacteria responsible for the properties of yogurt) regarding the production and use of formate. *Lactobacillus* cannot produce this metabolite, but it is essential for its proper development, unlike to *Streptococcus*, which can produce formate from pyruvate, an intermediate metabolite of the decomposition of lactose. This symbiotic relationship may be modified by the previous treatment of the milk. Thus, for instance, it was observed that an intense thermal treatment as used in the study (105 °C/30 min) yielded enough formate for the growth of the *Lactobacillus bulgaricus* strain used. The authors of the study found that the production of formate by *Streptococcus thermophillus* was redundant as there was sufficient in the milk due to the intensity of the treatment applied and, consequently, caused changes in the cooperation between the bacterial strains in terms of the production of metabolites, such as lactate, galactose, phenylalanine, and 3-hydroxyisobutyrate.

The composition of yogurt evolves during storage and a post-acidification stage begins in where the study of the changes produced in the metabolome could reflect the behavior of the starter cultures [79]. Metabolomics may provide information on the regulation of metabolites at this stage and used to monitor their evolution. In a yogurt made with goat’s milk, different regulations were counted for 39 metabolites of the 129 previously identified by GC-MS. During the first 14 days of storage, the metabolic pathways for the biosynthesis of aminoacyl-tRNA, phenylalanine, tyrosine, and tryptophan showed alterations, while the metabolism of fatty acids and propanoate was altered during the second half of storage, between days 14 and 28. In the same study, a metabolite-gene interaction analysis identified genes regulated by differentially expressed metabolites [80]. Deciphering the mechanisms of alteration in the production of metabolites at any point in the process may aid in the more meticulous control of the stages to yield a higher quality product. In the case of freeze-dried milk, Zhu et al. [81] reported that storage temperatures below 5 °C may protect the product from variations in its metabolomic profile. A multiplatform approach carried out by NMR spectroscopy and UHPLC-QTOF MS was used for the comprehensive mapping of the powdered milk metabolome, detecting variations in compounds, such as orotic acid, riboflavin, acetyl-carbohydrate, threonic acid, uridine, and various free fatty acids.

#### Study of the Behavior of Microorganisms during the Fermentation and Maturation Stages

Microorganisms play a prominent role in the final properties of some of the most widespread dairy products. The fermentation processes provide the characteristic aroma, taste, and texture of products, such as yogurt, kefir, or sour milk, defining them within the wide market of dairy derivatives. In the same way, the maturation process in cheese depends on the interaction of the different individuals that compose its microbiota, as if it were a community of neighbors. This microbiome is especially complex in milk and its derivatives, being also subject to variations due to the influence of the external conditions, where parameters, such as temperature, water activity (aw), and pH, among others, can play a determining role in the final characteristics, affecting the quality directly [82]. Omic approaches may provide a superior understanding of the microbial ecosystem [83] and intervene in the design on the production stages.

Transcriptomics has contributed to the attempt to fully study the behavior of the bacterium *Lactococcus lactis*, commonly used as a starter culture in cheese production. This lactic acid bacterium faces a stressful environment during cheese ripening, contributing much of the volatile compounds responsible for its flavor. The analysis of the proteolytic transcriptome of different species of lactic acid bacteria during the ripening of a cheese made with raw ewe’s milk exposed the dynamics of specific genes responsible for proteolytic reactions and showed the great influence of lactococci in this degradation process. In addition, the same study provided information on the role and properties of the genus *Lactobacillus*, confirming the activity of genes related to proteolysis during cheese ripening [84]. The results achieved are a clear example of how metagenomics and metatranscriptomics (study of the transcriptome of a biologic community) can be used together successfully in the analysis of the dairy microbiome. A multi-omic approach with the same combination of omic techniques served for the in-depth study of the behavior of the microbial community in a Swiss-type Maasdam cheese, presenting *Lactococcus lactis* as the strongest and most representative bacterium, accounting for 80–90% of the total. The data obtained from the metatranscriptomic analysis of this species displayed upregulation of genes linked with the central metabolism, including vitamin biosynthesis and homolactic fermentation, during the maturation stage in cold room. On the other hand, genomic analysis revealed the presence of alternative pathways to produce various compounds affecting the flavor, such as methanethiol, acetoin, diacetyl, acetate, or propionate [85]. Other bacteria involved in milk fermentation have also been recently analyzed at the transcriptome level, such as *Lactobacillus delbrueckii* subsp. *bulgaricus* and *Streptococcus thermophilus* (starter cultures in yogurt production) (Table 2).

Although the study of the transcriptome facilitates a better understanding of the bacterial machinery, proteomics and metabolomics are the omic techniques that show the corresponding final products. In particular, metaproteomics (study of the proteome of a biologic community) can provide a huge amount of information on how the set of enzymes of a microbial community act. The application of UPLC-ESI MS/MS on a kefir outlined the peptides released from milk proteins during fermentation, enabling the monitoring of proteolytic reactions and the delivery of digestion patterns. A maximum of 2828 different peptides were found to belong to 22 protein annotations, with a maximum degree of release in the first 24 h of fermentation [86]. A shotgun approach using label-free quantification described the proteome of several *Lactococcus lactis* strains, showing a profile composed by 586 proteins, which are suspected to be related to the resistance of these bacteria to different stress conditions. The results revealed that the proteins involved in the translation process were the most abundant. Interestingly, a subset of conserved proteins was found unique to some members of the *Lactococcus lactis* species, such as *L. lactis* subsp. *cremoris* and *L. lactis* subsp. *lactis*. These findings provide important knowledge about the core proteome of these bacteria, very important from the biotechnological point of view to improve the understanding of cell functions and thus optimize their use in bioprocesses, mainly as starter cultures in fermentation stages [87].

The analysis of data from the combination of transcriptomic, proteomic, and metabolomic techniques in the same study increases the amount of information to be processed and therefore the probabilities of a better understanding of the phenomena that occur during microbial metabolism in the transformation of dairy products. Such scenario is not yet frequent in the foodomics of dairy foods, but some recent studies are trying to open this window towards research focused on multi-omics. Qiao et al. [88] used transcriptomic and proteomic technologies aimed at examining changes at the transcription and protein level during the growth of *Streptococcus thermophilus* in fermentations at controlled pH. During the process, the bacterium differentially expressed up to 1396 genes and 876 proteins. The most significant changes occurred in the late lag phase and were associated with heterofermentation, glycolysis, peptidoglycan biosynthesis, conversion between amino acids, and stress response. The study offers a theoretical base for the optimization of media and bioprocesses during high-density culture of *Streptococcus thermophilus*. Similarly, proteomics was used in parallel with metabolomics in the characterization of metabolic activities of different strains belonging to *Lactobacillus delbrueckii* subsp *bulgaricus* and *L. delbrueckii* subsp *lactis*, providing data on their divergent origin and describing differentiated metabolic pathways for folate, amino acids, and sugar. Other metabolites, such as fatty acids and organic acids (e.g., lactate, acetate, and formate) were also detected and quantified [89]. More recent studies published that used metabolomic analysis to characterize the activity of microorganisms related to dairy biotechnology are displayed in Table 2.

**Table 2:** Application of omic technologies in the study of fermentation processes for the improvement of dairy products.

Dairy Product or Culture Medium	Microorganism/s Involved	Omic/s Technologies	Applied Technologies and/or Techniques	Highlighted Findings	Reference
M17 broth	*Streptococcus thermophilus* TH1436 and TH1477	Transcriptomics	RNA-sequencing	Overexpression of genes related to acid fermentation, phosphotransferase system, sugar transporter, and stress response in Gal^+^ species and underexpression of genes related to amino acids, protein metabolism, and CRISPR associated proteins in Gal^−^ species. Modification of the metabolism in Gal^+^ strains depending on the environment.	Giaretta et al. [90]
Yogurt	*Lactobacillus delbrueckii* subsp. *bulgaricus* ATC11842	Transcriptomics	RNA-sequencing and quantitative real-time PCR	Genes poorly expressed between the end of fermentation and the beginning of storage seem to be related to the post-acidification stage. The overexpression of the LDB_RS05285 gene could be involved in the reduction of lactic acid without affecting the growth of the strain.	Zhang et al. [91]
Swiss-type Maasdam cheese	*Lactococcus lactis* subsp. *lactis*, *L. lactis* subsp cremoris, *Lactobacillus rhamnosus*, *Lactobacillus. helveticus*, and *Propionibacterium freudenreichii* subsp. *shermanii*	Genomics and transcriptomics	Not specified	*Lactococcus lactis* is the dominant species of the cheese microbiota. Different pathways for the formation of flavor and production of free fatty acids, acetoin, diacetyl, acetate, ethanol, and propionate. Reduced center metabolism during cold storage except for *Lactococcus lactis*.	Duru et al. [85]
Raw ewes’ milk-based cheese	LAB community	Genomics and transcriptomics	PCR	*Lactococcus* is the dominant group of bacteria in cheese. Information on the role and properties of members of the *Lactobacillus* and *Lactococcus* genera	Pangallo et al. [84]
Fermented designed medium	*Streptococcus thermophilus*	Transcriptomics and proteomics	SDS-PAGE and iTRAQ HPLC-TOF MS/MS	Significant changes in the expression of 1396 genes and 876 proteins during fermentation. Relevant changes in heterofermentation, glycolysis, peptidoglycan biosynthesis, conversion between amino acids, and stress response occurred in the late-lag phase.	Qiao et al. [88]
M17 broth	*Lactococcus lactic* subsp. *lactis* IL1403, *L. lactic* subsp. *lactis* NCDO2118, *L. lactic* subsp. *cremoris* MG1363, and *L. lactic* subsp. *cremoris* NZ9000	Proteomics	Label-free quantification nUPLC-nESI MS/MS	Probable involvement of the core proteome in resistance to stress and probiotic activity. The proteins related to the translation process are the most abundant in the core proteome. Presence of exclusive conserved proteins in some of the strains. Detection of a specific proteome in subsp. *lactis* NCDO2118.	Silva et al. [87]
Goat’s milk kefir	Kefir grains	Proteomics	UPLC-nESI MS/MS	Identification of 2238 unique peptides corresponding to 22 protein annotations. Maximum peptide release during the first 24 h of fermentation. Different digestion patterns according to the nature of the proteins. Identification of 11 peptides with recognized biological activity.	Izquierdo-gonzález et al. [86]
Fermented Elliker broth	*Lactobacillus delbrueckii* subsp. *Bulgaricus* ATCC11842, *L. delbrueckii* subsp. *lactis* LMG6401, and the isolate *L. delbrueckii* 23 from Mozzeralla di Bufala Campana	Metabolomics and proteomics	NMR spectroscopy (H-NMR analysis) and 2D LC-LTQ MS	Differences in the metabolic pathways of folate, amino acids, and sugar in the three strains. The origin of the strains is divergent. *Lactobacillus delbrueckii* subsp. *Bulgaricus* ATCC11842 showed probiotic properties.	Zanni et al. [89]
GABA-rich cheese	GABA-producing bacterial strain *L. lactis* subsp. *Lactis* biovar diacetylactis 01-7 and non-GABA-producing bacterial strain *L. lactis* subsp. *cremoris* 01-1	Metabolomics	HPLC, LC-ESI MS/MS, and LC-LTQ Orbitrap MS	Presence of GABA and ornithine in cheese fermented with GABA-producing bacterial strain. Presence of citrate in the control cheese. Higher presence of peptides with antihypertensive activity and other functions in GABA-rich cheese. Lower amount of YL peptide in the GABA-rich cheese compared to the control.	Hagi et al. [92]
Yogurt	*Lactobacillus delbrueckii* subsp. *Bulgaricus* and *Streptococcus thermophillus*	Metabolomics	UPLC-Triple TOF MS/MS	Increase of 45 metabolites and decrease of another 47. Strict anaerobic fermentation promotes metabolic changes in bacteria and nutritional changes in yogurt.	Ding et al. [93]
Yogurt	*Lactobacillus delbrueckii* subsp. *Bulgaricus*, *Streptococcus thermophillus*, *lactobacillus plantarum* Taj-Apis362 UPMC90 and *lactobacillus plantarum* Taj-Apis362 UPMC91	Metabolomics	NMR spectroscopy (H-NMR analysis)	Probable influence of *Lactobacilus* strains UPMC90 and UPMC91 on the metabolic profile. The addition of glucose increases the production of GABA by the UPMC90 and UPMC91 strains. GABA is produced by *Lactobacillus delbrueckii* subsp. *Bulgaricus* and *Streptococcus thermophillus* without the addition of glucose. Lower lactose content in GABA-rich yogurt.	Hussin et al. [94]
Fermented skim milk	*L. plantarum* P9	Metabolomics	UPLC-QTOF MS/MS	Significant change in the metabolome after fermentation and cold storage (increase of 25 metabolites and decrease of another 10). These metabolites include fatty acids, peptides, and carbohydrates. Some of them could contribute functional attributes to the fermented product.	Zha et al. [95]

SDS-PAGE: sodium dodecyl sulphate polyacrylamide gel electrophoresis; iTRAQ: isobaric tags for relative absolute quantification; HPLC: high-performance liquid chromatography; TOF: time-of-flight; MS: mass spectrometry; MS/MS: tandem mass spectrometry; nUPLC: nano ultra-performance liquid chromatography; nESI: nano electrospray ionization; NMR: nuclear magnetic resonance; H-NMR: proton nuclear magnetic resonance; LC: liquid chromatography; LTQ: linear trap quadrupole; QTOF: quadrupole time-of-flight; Gal^+^: galactose-positive; Gal^−^: galactose-negative; CRISPR: clustered regularly interspaced short palindromic repeats. Some technologies and/or techniques used could have been missed by not expressly referring to them in the corresponding studies.

## 4. Multi-Omics. The New Horizon for ‘Omic’ Technologies

Throughout this review, the technologies of transcriptomics, proteomics and metabolomics have been shown to be beneficial in the understanding and characterization of dairy products at the biological level. However, the real power of these technologies is achieved when they are used in combination. Integrated approaches allow the combination of individual omic data sequentially or simultaneously, contributing to assess the information flow from one omic level to another [96]. The characterization of microbial communities through the application of metagenomics is a promising and powerful technology capable of safeguarding food safety, but its combination with other technologies such as transcriptomics, proteomics and metabolomics, would favor an even deeper identification and characterization of the microbial communities present in food [97], with data on phenomena at the level of gene expression, protein formation, and metabolic production. A binomial approach to these technologies has recently been tested during the fermentation processes in different food matrices shown in the present review (Table 2). Although approaches of this caliber are not yet common, other examples of the use of these multilevel platforms can be found in recent literature. Thus, Bellasi et al. [98] positively verified how the combinatorial use of metabolomics and metagenomics can provide a comprehensive understanding of the chemical composition and properties of cow’s milk. The exhaustive analysis of these milk characteristics provided the authors with knowledge on which to rely when selecting the most suitable raw material in the elaboration of a product such as cheese.

Despite the potential demonstrated by these approaches, their use is still largely limited to the prohibitive cost of some methods. On the other hand, other important challenges still remain to be faced, such as the computational treatment of the data sets obtained, which may reach truly exorbitant sizes [99]. This heterogeneous data set, which can come from a variety of sources, equipment, and experimental settings, needs to be processed, normalized, and integrated. In short, being transformed into understandable information [100]. Unfortunately, researchers are limited in accessing complete data records or in identifying multi-omic data sets that alleviate their concerns during the research process. Today, there is no unified public repository for multi-omic data because most of them are created for each study, with specific technologies. Therefore, these data repositories are prepared according to the type of omic technology used and the type of assay. This lack of public infrastructure has enabled the creation of cloud-based hosting and analysis platforms, such as Lifebit or Steven Bridges Genomics. In addition, software applications such as STATegraEMS8 have also been released to address this type of issues [101].

Another disadvantage of this technology is the susceptibility of the data to different sample preparation methods, which establishes undesirable biases [102]. However, as with all new technology, difficulties and disadvantages are gradually resolved. Based on the recent advances observed in this field, it would not be surprising that in the medium long term the multi-omic approach was a reference in food analysis, being used in routine tests by laboratories.

## 5. Conclusions

Foodomics led by transcriptomics, proteomics, and metabolomics is proving to be a multidisciplinary area of knowledge capable of providing solutions to improve the quality of dairy products in terms of the fight against food fraud and the maintenance of food safety. Obtaining protein and metabolic biomarkers is an effective strategy for detecting undeclared changes in dairy products, allowing the information presented on the label to be contrasted. In this regard, the MALDI-TOF MS technique can provide a lot of information in relation to different factors, such as the presence of foodborne pathogens or the origin of milk. Additionally, transcriptomic, proteomic, and metabolomic technologies are revealing lots of information about the events that occur at the molecular level in dairy products during their transformation, helping to understand the impact that factors, such as thermal treatment, storage, and fermentation or maturation processes, have on the final features of the food. In short, these omic approaches and especially their combination has immense potential for the improvement of dairy products and their evaluation in terms of safety and consumer protection, the inclusion of which as routine techniques could be a reality in the coming years.

## Figures and Tables

**Table 1:** Example of the use of the MALDI-TOF MS technique for fraud detection and authenticity assurance in cheeses.

Aim of the Study	Type of Cheese	Biomarkers Found	Reference
Identify the use of frozen milk in cheese production	Mozzarela di Bufala Campana	GLYCAM1-derived phosphopeptides	Arena et al. [49]
		β-casein-derived phosphopeptides	
		γ-Casein/β-casein peptides	
		α-Lactalbumin peptides	
		β-Lactoglobulin peptides	
		Unknown peptides	
Identify the origin and presence of cow’s milk in a cheese made of water buffalo milk	Italian water buffalo Mozzarella cheese	α_s1_-Casein peptides	Aira et al. [50]
		β-Casein peptides	
Define the original geographical location and PDO of a traditional cheese	Coalho cheese	α_s1_-Casein peptides	Fontenele et al. [51]
		α_s2_-Casein peptides	
		β-Casein peptides	
		β_A2_-Casein peptides	
		β_A3_-Casein peptides	
		κ-Casein peptides	
Identify the species of milk used in making cheeses	Feta and Mozzarella cheeses	Not specified	Rau et al. [52]
Authenticate and discriminate different cheeses	Korean Mozzarella cheese	α_s1_-Casein peptides	Kandasamy et al. [53]
		β-Casein peptides	
		α_s2_-Casein peptides	
		κ-Casein peptides	
		Unknown peptides	
Identify the species of milk used in making cheeses	Feta cheese	Not specified	Kritikou et al. [54]

GLYCAM1: glycosylation-dependent cell adhesion molecule 1; PDO: protected designation of origin.
